# Evolving Profile of HPV-Driven Oropharyngeal Squamous Cell Carcinoma in a National Cancer Institute in Italy: A 10-Year Retrospective Study

**DOI:** 10.3390/microorganisms8101498

**Published:** 2020-09-29

**Authors:** Maria Gabriella Donà, Francesca Rollo, Barbara Pichi, Giuseppe Spriano, Silvia Moretto, Renato Covello, Raul Pellini, Maria Benevolo

**Affiliations:** 1 STI/HIV Unit, San Gallicano Dermatologic Institute IRCCS, Via Elio Chianesi 53, 00144 Rome, Italy; mariagabriella.dona@ifo.gov.it; 2 Pathology Department, Regina Elena National Cancer Institute IRCCS, Via Elio Chianesi 53, 00144 Rome, Italy; renato.covello@ifo.gov.it (R.C.); maria.benevolo@ifo.gov.it (M.B.); 3 Otolaryngology Head and Neck Surgery Department, Regina Elena National Cancer Institute IRCCS, Via Elio Chianesi 53, 00144 Rome, Italy; barbara.pichi@ifo.gov.it (B.P.); silvia.moretto@ifo.gov.it (S.M.); raul.pellini@ifo.gov.it (R.P.); 4 Department of Otolaryngology Head and Neck Surgery, Humanitas Clinical and Research Center—IRCCS, via Manzoni 56, 20089 Rozzano, Milan, Italy; giuseppe.spriano@hunimed.eu

**Keywords:** neoplasms, oropharyngeal squamous cell carcinoma, human papillomavirus, HPV-induced neoplasms, p16

## Abstract

Globally, 30% of oropharyngeal carcinomas (OPSCC) are caused by Human Papillomavirus (HPV). Recently, increasing incidence trends for HPV-driven OPSCC have been reported in many countries and changes in the typical HPV-positive OPSCC patient have been recorded, with an increase in the median age and diagnoses in women. We investigated the characteristics of the OPSCC patients attending an Italian Cancer Institute from 2010 to 2019 and assessed possible changes overtime of demographic, behavioral, and clinico-pathologic variables of HPV-driven OPSCC. Overall, 339 OPSCCs were evaluated. HPV-DNA and p16 positivity were 48.7% and 55.2%, respectively, with an HPV-driven fraction (i.e., HPV-DNA+/p16+) of 48.3%. We observed a significant increase overtime in the rate of HPV-associated cases (53.7% in 2015–2019 vs. 40.3% in 2010–2014, *p* = 0.019). The rate of HPV-driven cases was significantly higher among women, never smokers, patients with T1–T2 tumors, and with nodal involvement. A trend was also observed toward an increase in HPV-driven OPSCCs among patients >61 years, women, former smokers, and patients with no nodal involvement in 2015–2019. Our findings consolidate the observation that HPV-associated OPSCCs are also increasing in Italy. Moreover, they suggest that the profile of the HPV-driven OPSCC patient might be changing.

## 1. Introduction

Head and neck squamous cell carcinomas represent the sixth most common malignancy by incidence worldwide and are mostly caused by tobacco and alcohol consumption [1]. However, oral, laryngeal and particularly oropharyngeal cell carcinomas (OPSCC) have also been associated with Human Papillomavirus (HPV) infection. Overall, 42,000 OPSCC cases worldwide were caused by HPV in 2018, corresponding to an HPV-attributable fraction of 30% [2]. However, a significant geographic variation in the HPV-attributable fraction exists, with the highest prevalence found in North America, Northern and Central Europe [3,4,5]. Despite this heterogeneity, HPV16, which has been recognized as a causative agent of OPSCC by the International Agency for Research on Cancer (IARC) [6], represents the most prevalent genotype in HPV-related OPSCCs worldwide.

The role of HPV in the development of OPSCC has led to essential modifications both in the Classification [7] and in the staging system of these neoplasias [8]. Importantly, HPV-negative and -positive OPSCCs present distinct epidemiologic profiles, especially regarding age at onset and association with tobacco and alcohol consumption [9]. They also differ in terms of pathologic features (e.g., tumor size and nodal involvement). Notably, patients with HPV-driven cancers display a significantly improved prognosis and response to therapy, irrespective of treatment modality [10], compared to those with HPV-negative tumors mainly associated with reduced loco-regional failure, and different patterns and timing of distant metastases for HPV-positive patients [11,12,13,14]. Compared to HPV-negative patients, those with an HPV-driven tumor have an 80% decreased 5-year risk of progression and death [15]. Nevertheless, it should be noted that up to 25% of HPV-positive tumors do not show these favorable prognostic characteristics and can relapse within two years of diagnosis [12,16], indicating the need to improve patient stratification, which is currently based only on the presence of the HPV surrogate marker, p16. The latest World Health Organization (WHO) Classification of Head and Neck Tumors underscores the importance of direct HPV testing. Detection of HPV-DNA, in combination with p16 and/or HPV-mRNA evaluation, represents one of the most reliable diagnostic approaches for the identification of HPV-driven OPSCCs [17].

In recent decades, increasing incidence trends for HPV-driven OPSCC have been reported in many countries, particularly the USA [18,19,20,21,22] possibly as a result of changes in sexual behavior starting from the 50s, which led to increased HPV exposure [23]. Similar trends have been reported for Northern European countries [24,25,26,27]. Moreover, changes in the typical HPV-positive oropharyngeal cancer patient have been recorded, with an increase in the median age at diagnosis [23,28,29], and an increase in the proportion of women [30]. However, most of the available data on HPV prevalence in OPSCCs and on the features of HPV-positive cancer patients derive from studies conducted in the US and Northern Europe. Conversely, there are sparse data concerning Southern Europe and only a few studies, with small sample sizes, have been conducted in Italy [3,31,32]. In a previous study of our group, we assessed the HPV-attributable fraction in a series of cases diagnosed at the Regina Elena National Cancer Institute between 2010 and 2014. We found that 39.8% of the OPSCCs were HPV-associated [33]. In this study, we aimed to investigate whether the HPV-attributable fraction and the epidemiologic profile of OPSCC patients have changed in the following five years. To this end, we assessed HPV-DNA prevalence, p16 over-expression and genotype distribution in all the consecutive primary OPSCC cases over the last decade. On this larger series, we evaluated which variables the HPV etiology is associated with and whether, and how, changes over time occurred.

## 2. Materials and Methods

### 2.1. Study Population

The study included all consecutive cases with a histologically confirmed diagnosis of primary and untreated OPSCC (tonsils, base of tongue, soft palate, uvula, Waldeyer ring, and other parts of the oropharynx, based on the International Classification of Diseases-10 codes for the oropharynx) that were tested for HPV-DNA on formalin fixed paraffin embedded (FFPE) tissue at the Pathology Department of the Regina Elena National Cancer Institute between June 2010 and December 2019.

For each patient, the medical records were used to retrieve data regarding gender, age at diagnosis, history of tobacco use, primary tumor subsite, and histopathologic factors. Missing data were due to incomplete medical records. Patients provided a general consent for use of their personal and clinical data for research purposes. The study was approved by the Ethics Committee of the Regina Elena National Cancer Institute (CE/485/12; 2012/10/18).

### 2.2. HPV-DNA Testing

HPV-DNA detection and genotyping were performed using the Inno-LiPA HPV Genotyping Extra or Extra II kit (Fujirebio, Pomezia, Italy), for the cases diagnosed until 2014 and since 2015, respectively. Both assays are based on the SPF10 primer set used for the amplification of a 65-bp fragment within the L1 region and they detect individually, among others, the high-risk HPV 16, 18, 31, 33, 35, 39, 45, 51, 52, 56, 58, and 59, the probably high-risk HPV68, and the possibly high-risk HPV66 [6]. The protocol for FFPE block sectioning and processing for HPV testing has been previously described [33].

### 2.3. p16 Immunostaining

Immunohistochemical staining for p16 was performed using the CINtec^®^ Histology kit (Roche Diagnostics, Milan, Italy), following the manufacturer’s instructions. The immunostaining, which was independently evaluated by two investigators blinded to the HPV findings, was considered as positive when a strong nuclear expression was observed for >75% of tumor cells [8].

### 2.4. Statistical Analyses

Descriptive statistics were used to summarize the characteristics of the study population (mean and range, median and Interquartile Range, IQR, frequency). Samples containing any of the 12 high-risk HPVs were considered positive for high-risk types. To analyze the HPV-attributable fraction, only the cases evaluated for both HPV-DNA and p16 staining were included in the analysis. They were considered as HPV-driven when positive for both markers. In order to investigate changes over time, two periods of diagnosis of approximately the same length were taken into account: June 2010–December 2014 (*n* = 146 OPSCCs) and January 2015–December 2019 (*n* = 193 OPSCCs). Analyses were performed both overall and according to the period of diagnosis. The following variables were included: age at diagnosis (higher or lower than the median age of patients, i.e., 61 years), gender, smoking habits (never, former and current smoker), cancer subsite (tonsils, base of the tongue, or other oropharyngeal sites), tumor classification (T1–T2 vs. T3–T4) and nodal classification. In order to overcome discrepancies due to changes in pathologic classification that had taken place during the study period, we classified the nodal status in two groups: patients with (N+, independently of number and size of the lymph nodes involved) and without nodal involvement (N0). A chi-square test was used to test the relationship between each variable and the outcome of interest (i.e., HPV-driven OPSCC). The Mann-Whitney test was used for comparisons between median values. A *p* value < 0.05 was considered as statistically significant. Statistical analyses were conducted using the MedCalc Statistical Software v. 19.3.1 (MedCalc Software Ltd., Ostend, Belgium).

## 3. Results

Due to the emerging data regarding a change in the HPV-attributable fraction and in the epidemiologic profile of OPSCC patients in developed countries, we aimed to evaluate whether similar modifications are also occurring in an Italian population sample. To this end, 339 OPSCC cases, diagnosed over a 10-year period, were included in this study. We noticed a slight but not significant increase in OPSCC diagnosis over time (2010–2014: 32.4 cases/year; 2015–2019: 38.4 cases/year, data not shown). Table 1 summarizes the distribution of patient characteristics, overall and in the two study periods.

The median age for the overall population was 61 years with no significant modification over time. In the two study periods, we evidenced no substantial modification in the distribution of cases by cancer subsite, T, N and smoking status, whereas we evidenced an increase, although marginally significant, in OPSCC diagnosis among women (25.9% vs. 17.1%, *p* = 0.054).

Table 2 shows the HPV-based marker distribution, overall and according to the period of diagnosis.

Overall, the HPV-DNA and p16 positive cases were 48.7% and 55.2%, respectively, with an HPV-driven fraction of 48.3%. When we considered the HPV-DNA results or the combination of HPV-DNA and p16 over time, we observed a significant increase in the rate of positive cases in the second period compared to the first one (*p* = 0.015 and *p* = 0.019 respectively), while the increase of p16 positivity showed only a trend towards statistical significance (*p* = 0.06).

**Figure 1:**
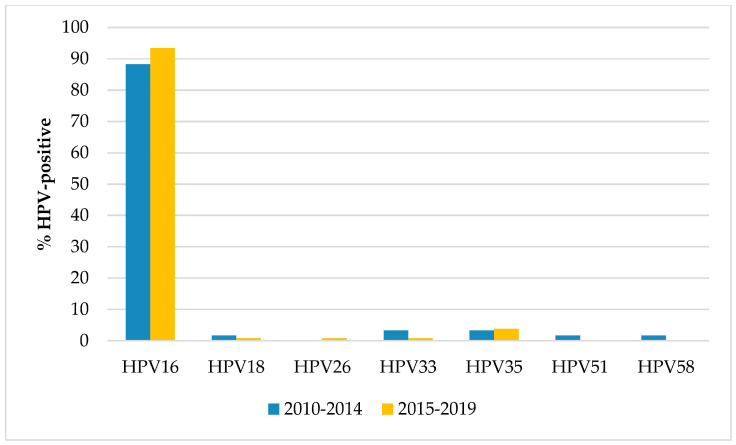
Prevalence of the individual HPV genotypes among the HPV-positive oropharyngeal squamous cell carcinomas according to the period of diagnosis.

Regarding the genotype distribution (Figure 1), HPV16 was the most prevalent type overall (91.5% of the HPV-positive OPSCCs) and in each period, followed by HPV35 (overall prevalence 3.6%), with no significant differences over time. In the first period, HPV33 showed the same prevalence of HPV35 (3.3%) while in the second period no other genotype exceeded a prevalence of 1%.

We then analyzed the association between the HPV-driven fraction and the variables under study, overall and in each time period (Table 3).

**Figure 2:**
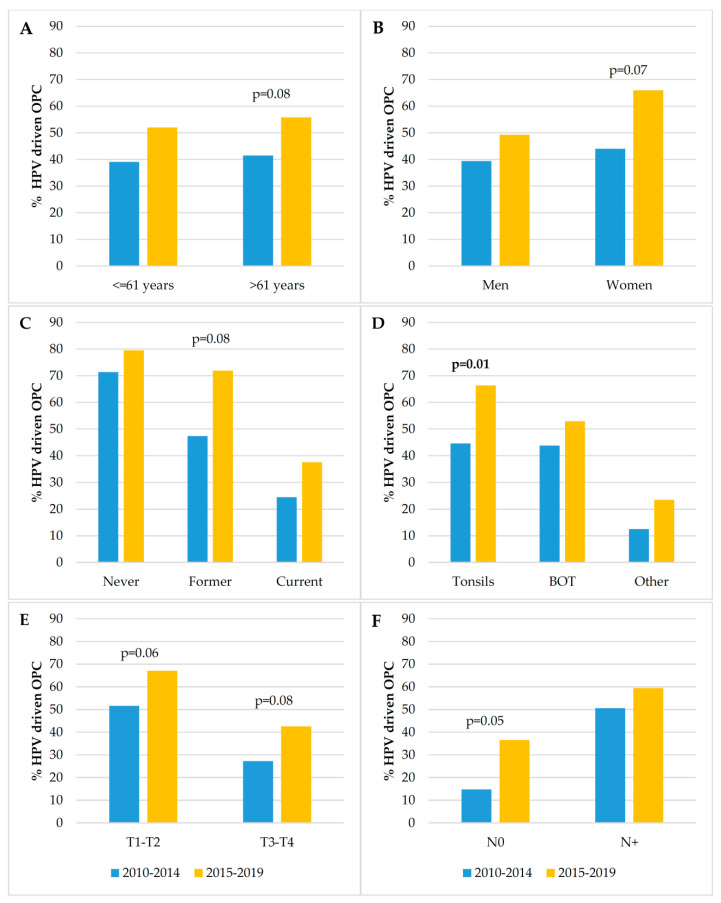
Prevalence of HPV-driven oropharyngeal squamous cell carcinomas (OPSCC) according to the selected variables and the two periods of diagnosis: (**A**): age at diagnosis; (**B**): sex; (**C**): smoking status; (**D**): cancer subsite (Tonsils; BOT, base of the tongue; Other, soft palate, uvula, epiglottic vallecula, amygdaloglossus sulcus, posterior wall); (**E**): T status (primary tumor category); (**F**): N status (N0, without nodal involvement; N+, with nodal involvement); *p* values for the comparison between periods are only shown when <0.10. Significant differences are highlighted in bold (cut-off for statistical significance was set at 0.05).

HPV-driven tumor distribution did not change with age at diagnosis. Moreover, the median age of patients with an HPV-driven OPSCC did not change significantly over time (62 years, IQR: 55–70 in 2010–2014 vs. 61 years, IQR: 55–68 in 2015–2019, *p* = 0.83; data not shown). HPV-related cases were more prevalent in women, although the difference by sex was not significant in the first period. Moreover, overall and in each of the two periods, the HPV-driven rate was significantly associated with smoking status, the highest prevalence being observed among never smokers, with T status, being higher in smaller tumors, and with N status, being higher in those with positive lymph-node status. In addition, HPV-driven OPSCC prevalence was significantly associated with cancer subsite when taking into account all the ten-year observation period and in the most recent years, while only a trend towards statistical significance was evidenced in the previous period. Tonsils were always found to be the site with the highest prevalence of HPV-driven cases. Furthermore, we compared the prevalence of the HPV-driven fraction between the two periods according to the variables under study in order to investigate whether changes have occurred over time (Figure 2). We observed a significant difference only for OPSCC arising in the tonsils, with a significant increase in the HPV-driven fraction in 2015–2019. Trends of increase in the HPV-driven fraction in the most recent period were observed for patients aged >61 years, women, former smokers, T1–T2 and also T3–T4 tumors, and for cancers with no nodal involvement.

## 4. Discussion

In this study, we investigated the characteristics of the OPSCC patients that were tested for HPV at the Regina Elena National Cancer Institute in the last 10 years. We also assessed possible changes over time of demographic, behavioral, clinical and pathologic variables of HPV-driven OPC, selecting the variables identified as predictors of survival for OPSCC patients.

Overall, a higher number of cases was observed in the most recent period (193 in 2015–2019 vs. 146 in 2010–2014). The most up-to-date overview of OPSCC incidence trend in our country has reported an increasing trend over the period 1998–2010 [34]. However, there is little or no recent data regarding this OPSCC trend. In the absence of such data, we may reasonably speculate, however, that the increasing contribution of HPV to OPSCC on the one hand (see below), and the continuing heavy tobacco and alcohol exposure in our country on the other hand, have caused an increase in OPSCC incidence also after 2010. Our observation might thus reflect a recent national tendency. The increase in the number of diagnoses might be also due to a random fluctuation. However, given that our Institute is rapidly becoming a point of reference at national level for this specific pathology, which requires a multidisciplinary approach, this is possibly one of the reasons why we have registered an increasing number of cases.

OPSCC diagnoses were more frequent in men than women, with a M/F ratio of 3.5, in line with the global scenario of a higher burden of this neoplasia among men. Globally, OPSCC diagnoses in men are 4 times more frequent than in women [2]. OPSCC cases diagnosed in the two time periods were similar regarding all the selected variables, except for sex distribution and T status. Regarding sex distribution, despite the fact that the majority of OPSCC patients were males in both study periods, we observed a marginally significant increase in the diagnoses among women in 2015–2019. An increase in OPSCC diagnosis among women could have a positive impact on survival of OPSCC patients, given that it has been reported that being female is associated with a marked improved survival after an OPSCC diagnosis (irrespective of HPV status) in Europe [35].

Concerning T status, we found no significant difference between the two periods, even though we observed an increase in OPSCCs with T3–T4 status in 2015–2019 compared to 2010–2014. This difference may be due to the small number of cases and/or missing data especially in the first period.

Overall, the median age of our OPSCC patients was 61 years, and remained similar in the two periods, with no significant changes observed. A slightly higher median age was observed for OPSCC patients diagnosed in a large clinical center in Italy, i.e., 65 years [32]. Similarly, distribution of cancer subsites showed no change over time, tonsillar carcinoma being the most frequent OPSCC in both periods. The Italian study mentioned above also found that the tonsils were the most frequently involved cancer site.

Overall, HPV-driven cases were approximately 48%. This figure is lower than those found in Northern European countries, such as Sweden and Denmark, where the HPV-attributable fraction is well over 60%, but it is still high when compared to other Southern European countries [2,5,36] and other Italian series of OPSCC cases [32]. The lower HPV prevalence in Southern compared to Northern Europe may be due to different behavioral factors, such as sexual and smoking habits. Indeed, smokers are much more common in Italy than in Northern Europe so the role of HPV in OPSCC development is possibly diluted by tobacco habit, which is still relevant in our country. Notably, we observed a significant increase in HPV-DNA and marginally significant increase in p16 positivity over time. Most importantly, a significantly higher HPV-attributable fraction was found in the more recent period (53.7% vs. 40.3%; *p* = 0.019). These findings confirm other European [2,37] and Italian data [32], which indicate a marked increase in HPV-driven cases among OPSCC patients in recent years.

Regardless of the period of diagnosis, HPV16 represented the most frequent type detected in HPV-positive cases, with other high-risk types infrequently found, in line with the worldwide scenario for HPV-related OPSCCs [3]. Indeed, this result is predictable because although HPV16 elicits a humoral response to early virus proteins, especially E6, it is the most efficient genotype in evading the host immune system. This may be of utmost importance for tumors arising in the tonsils, which are a part of this system and may confer an advantage to HPV16 compared to other high-risk HPV types [38].

With regard to the patients with HPV-driven OPSCC, their median age at diagnosis was 61 years, in line with other countries. In the US, HPV-driven OPSCC diagnoses peaked in those aged 60–64 years [39]. We observed no significant change over time in the age of patients with HPV-driven cancers, differently from other recent studies. Thompson et al. observed a significant increase in the mean age at diagnosis as well as in the proportion of patients >65 years in the most recent years (2011–2016 vs. 2002–2010) [29]. This shift toward an older age for patients with HPV-related OPSCC, and in general with OPSCC, observed in the US [18,23,29] might be due to the aging of those involved in the sexual revolution. Interestingly, a study showed that increased HPV exposure among US women can be explained by changes in sexual behavior around the years of the sexual revolution, and, in turn, these changes parallel the increasing incidence of HPV-related cancers, included OPSCC [40]. However, since the sexual revolution was not the same in Europe, we may not necessarily expect a similar change in the age of patients with HPV-driven OPSCC [31].

Besides the increase in the overall OPSCC diagnoses among women, we also observed that the proportion of HPV-driven cases among women was significantly higher than that among men, overall and in 2015–2019. In the latter period, for every 10 OPSCC diagnoses in men, 5 were HPV-driven, whereas among 10 diagnoses in women, nearly 7 were caused by HPV. In the international study by Castellsaguè et al., which analyzed over 1,000 OPSCC cases, the HPV-attributable fraction was almost double in women compared to men [3]. Similarly, in the case series from the North of Italy, HPV-driven cases were even two times more frequent in women than men [32]. In addition, a recent study based on the available Italian cancer registries evidenced an increasing incidence of HPV-related head and neck cancers in females in 1988–2012, while a stable incidence in males was found [31]. Besides Italy, other European countries have observed increasing trends of HPV-associated OPSCC for women and not for men [41,42]. Indeed, Combes et al. evidenced that in some European countries, ours included, the M/F ratio of HPV prevalence in OPSCC is below 1, and suggested that this is due to the higher prevalence of smoking among men in these countries [43]. Differently, in Northern European countries, and in particular in the US, the M/F ratio is >1, and, according to the authors, differences in the fraction of HPV-attributable OPSCC by gender and country are consistent with variations in smoking habits, although other risk factors, such as sexual behavior and alcohol consumption need to be taken into account. In our study, the higher prevalence of HPV-driven cases in women compared to men may be explained by less frequent/heavy smoking habits among women, with a consequentially lower smoking-associated etiology for OPSCC arising in women in our country. Indeed, the lower prevalence of tobacco smoking in women compared to men in Italy is confirmed by WHO report [44]. Moreover, HPV prevalence in OPSCC female patients may be increasing because of the reduction in smoking among women in recent years, although in our study, this hypothesis cannot be verified because of the small number of women involved.

Regarding smoking status, HPV-driven OPSCC cases were significantly more common among never smokers, both overall and in each study period. The lowest prevalence of HPV-related cases was found in current smokers, in line with other studies [32,45,46]. When tobacco exposure occurs, this is likely the main cause of OPSCC, since only a minor fraction of cases diagnosed among smokers are HPV-driven. Although it is still unclear whether and how HPV and smoking interact in the development of head and neck cancer, it actually seems that HPV acts as a stronger carcinogen in non-smokers (i.e., in absence of a major risk factor) than smokers. It is also worth noting that smoking and HPV interplay is not only limited to their role in OPSCC etiology, but it also impacts on the outcome of patients with HPV-related OPSCC. A recent systematic review evidenced that smoking is associated with a worse overall survival in these patients [47].

When the proportions of HPV-attributable cancers in the two study periods were compared stratifying for each variable, the only significant change was found for cancer subsite. In detail, the HPV-driven cases among tonsillar cancers went from 44.6% in 2010–2014 to 66.3% in the following period. An increasing trend that did not reach statistical significance was observed for patients older than 61 years, women, former smokers, patients with T1–T2, T3–T4 and those with no nodal involvement. Specifically, we observed an increase over time in the prevalence of HPV-related OPSCCs in those over the age of 61 years (from 41.5% to 55.8% in the most recent period). The HPV-attributable fraction among women increased from 44.0% in 2010–2014 to 66.0% in 2015–2019, figures that nearly overlap those found in another Italian study, which evidenced a significant increasing trend from 46.2% (2007–2012) to 73.3% (2013–2018) [32]. Differently from the study by Del Mistro et al., which observed a significant change over time also for men, the increase we estimated for men was modest and did not reach statistical significance. Although referring to a less recent case series, a multi-national US study also observed a substantial increase in OPSCCs caused by HPV in women [13].

Regarding nodal status, we found an association, stable over time, between HPV etiology and nodal involvement. Indeed, it has been reported that patients with HPV-driven OPSCCs are more likely to have cervical lymphadenopathy, but nodal involvement does not have the same bad prognostic implications observed for HPV-negative OPSCC [38,48]. Interestingly, we noticed an increase, although marginally significant (*p* = 0.05), in the HPV-attributable fraction among the patients with no nodal involvement in the second period. These data need confirmation on a prospective larger series of cases.

This study has some limitations. Firstly, the single-center design means we cannot generalize our findings since they may not reflect the overall Italian landscape of patients with HPV-driven OPSCC. However, the study was conducted at a National Cancer Institute which cares for patients coming from the center and also south of the country. Since Italy lacks a population-based cancer registry, case series from single institutions are important to collect data that would otherwise be lost. The second limit regards the fact that we used smoking status and not pack-years as the smoking variable, due to the absence of detailed information in this regard. In addition, information on this variable was missing for a certain proportion of patients, particularly from the first period. Finally, the sample size for each period may have limited the ability to observe significant associations or differences, resulting in only trends towards statistical significance for certain variables. Moreover, the ten-year study period may not be sufficient to observe significant differences.

## 5. Conclusions

In conclusion, our study confirmed the established association between cancer-associated variables (tonsil subsite, small T and nodal involvement) and patient characteristics (smoking status) with OPSCC with HPV etiology. Moreover, a significant increase in the HPV-driven cases over time was evidenced, overall and for tonsillar cancers. Finally, in the most recent years, a trend was observed toward an increase in HPV-driven OPSCCs also among patients above 61 years of age, women, former smokers, and patients with no nodal involvement compared to the previous period. Taken together, our findings consolidate the concept that HPV-associated OPSCCs are increasing also in Italy and suggest, at least in part, a change in the profile of the HPV-driven OPSCC patient.

## Figures and Tables

**Table 1:** Demographic and clinico-pathologic characteristics of the oropharyngeal squamous cell carcinomas (OPSCC) of the study population overall and according to the period of diagnosis.

	Overall	2010–2014	2015–2019	*p* Value ^1^
**Median age, years (IQR)**	61 (54–68)	62 (55–69)	61 (54–68)	0.51
**Mean age, years (range)**	62 (28–90)	62 (37–84)	61 (28–90)	0.37
**Sex distribution, *n* (%)**				0.054
Men	264 (77.9)	121 (82.9)	143 (74.1)	
Women	75 (22.1)	25 (17.1)	50 (25.9)	
Total	339 (100)	146 (100)	193 (100)	
**Smoking status**				0.76
never	63 (22.9)	23 (22.3)	40 (23.3)	
former	55 (20.0)	23 (22.3)	32 (18.6)	
current	157 (57.1)	57 (55.4)	100 (58.1)	
Total	275 (100)	103 (100)	172 (100)	
**Cancer subsite, *n* (%)**				0.22
Tonsils	155 (45.7)	66 (45.2)	89 (46.1)	
Base of the tongue	133 (39.2)	63 (43.2)	70 (36.3)	
Other^2^	51 (15.1)	17 (11.6)	34 (17.6)	
Total	339 (100)	146 (100)	193 (100)	
**T status, *n* (%)**				0.07
T1-T2	160 (52.6)	71 (59.2)	89 (48.4)	
T3-T4	144 (47.4)	49 (40.8)	95 (51.6)	
Total	304 (100)	120 (100)	184 (100)	
**N status, *n* (%)**				0.58
N0	71 (23.4)	30 (25.0)	41 (22.3)	
N+	233 (76.6)	90 (75.0)	143 (77.7)	
Total	304 (100)	120 (100)	184 (100)	

^1^ For the comparison between the two periods. ^2^ Soft palate, uvula, epiglottic vallecula, amygdaloglossus sulcus, posterior wall.

**Table 2:** HPV-DNA status, p16 positivity and HPV-driven OPSCC cases overall and according to the period of diagnosis.

	Overall	2010–2014	2015–2019	*p* Value ^1^
	*n* (%)			
**HPV-DNA**				0.015
**negative**	174 (51.3)	86 (58.9)	88 (45.6)	
**positive**	165 (48.7)	60 (41.1)	105 (54.4)	
**Total**	339 (100)	146 (100)	193 (100)	
**p16 staining**				0.06
**negative**	142 (44.8)	66 (51.2)	76 (40.4)	
**positive**	175 (55.2)	63 (48.8)	112 (59.6)	
**Total**	317 (100)	129 (100)	188 (100)	
**HPV-driven (HPV-DNA+/p16+)**				0.019
**no**	164 (51.7)	77 (59.7)	87 (46.3)	
**yes**	153 (48.3)	52 (40.3)	101 (53.7)	
**Total**	317 (100)	129 (100)	188 (100)	

^1^ For the comparison between the two periods.

**Table 3:** Prevalence of HPV-driven oropharyngeal squamous cell carcinomas (OPSCC) according to selected variables, overall and according to the period of diagnosis.

HPV-Driven OPSCC						
	Overall		2010–2014		2015–2019	
Variable	*n*/N (%)	*p* Value	*n*/N (%)	*p* Value	*n*/N (%)	*p* Value
**Age, years**		0.63		0.77		0.60
≤61	78/166 (47.0)		25/64 (39.1)		53/102 (52.0)	
>61	75/151 (49.7)		27/65 (41.5)		48/86 (55.8)	
**Sex distribution**		0.039		0.68		0.043
Men	109/242 (45.0)		41/104 (39.4)		68/138 (49.3)	
Women	44/75 (58.7)		11/25 (44.0)		33/50 (66.0)	
**Smoking status**		<0.0001		0.0009		<0.0001
Never	46/60 (76.7)		15/21 (71.4)		31/39 (79.5)	
Former	32/51 (62.7)		9/19 (47.4)		23/32 (71.9)	
Current	48/145 (33.1)		12/49 (24.5)		36/96 (37.5)	
**Cancer subsite**		<0.0001		0.053		0.0001
Tonsils	82/142 (57.7)		25/56 (44.6)		57/86 (66.3)	
Base of the tongue	61/125 (48.8)		25/57 (43.8)		36/68 (52.9)	
Other ^1^	10/50 (20.0)		2/16 (12.5)		8/34 (23.5)	
**T status**		0.0001		0.013		0.001
T1-T2	89/147 (60.5)		32/62 (51.6)		57/85 (67.1)	
T3-T4	52/138 (37.7)		12/44 (27.3)		40/94 (42.6)	
**N status**		<0.0001		0.001		0.010
N0	19/68 (27.9)		4/27 (14.8)		15/41 (36.6)	
N+	122/217 (56.2)		40/79 (50.6)		82/138 (59.4)	

^1^ Soft palate, uvula, epiglottic vallecula, amygdaloglossus sulcus, posterior wall.
